# Lateral approach for supraclavicular brachial plexus block

**DOI:** 10.4103/0019-5049.65364

**Published:** 2010

**Authors:** DK Sahu, Anjana Sahu

**Affiliations:** Department of Anaesthesiolgy, Jagjivanram Railway Hospital, Mumbai, India; 1 Department of Anaesthesiology, TN Medical College & BYL Nair Ch. Hospital, Mumbai, India

**Keywords:** Brachial plexus block, lateral approach brachial block, lateral supraclavicular block, supraclavicular block

## Abstract

A lateral approach described by Volker Hempel and Dr. Dilip Kotharihas been further studied, evaluated and described in detail in the present study. The aim of this study was to evaluate lateral approach of supraclavicular brachial plexus block, mainly in terms of successes rate and complication rate. The study was conducted in secondary level hospital and tertiary level hospital from 2004 to 2008. It was a prospective nonrandomized open-level study. Eighty-two patients of both sexes, aged between 18 and 65 years with ASA Grade I and II scheduled to undergo elective major surgery of the upper limb below the midarm, were selected for this new lateral approach of brachial plexus block. The onset and duration of sensory and motor block, any complications and need for supplement anaesthesia were observed. Success and complication rate were calculated in percentage. Average onset and duration of sensory and motor block was calculated as mean ± SD and percentage. Out of 82 patients, 75 (92%) have got successful block with no significant complication in any case.

## INTRODUCTION

The brachial plexus block for upper limb surgery has proved to be a safer and effective method of regional anaesthesia. But it is a common observation that surgeries on upper limb are still being performed mainly under general anaesthesia despite unanimous consensus toward regional anaesthesia, due to one or the other reasons. Various approaches have been described such as supraclavicular, interscalene, transscalene,[1] infraclavicular and axillary, but they all are associated with some technical difficulties, inadequate blocks and significant complications. The rate of conversion or supplementation with general anaesthesia from brachial block is quite high. Volker Hempel[2] has described the method for supraclavicular brachial plexus block, where longitudinal placement of the needle is done in relation to the brachial plexus from lateral to medial with a high success rate. Dr. Dilip Kothari[3] has described lateral approach of supraclavicular brachial plexus block associated with minimal complication and high success rate. We found the lateral approach relatively easy to perform and it has less complication. In this study, we have further studied, evaluated and described the lateral approach of supraclavicular block, mainly in terms of anatomical landmark, success rate and complication rate.

## METHODS

Eighty-two patients of both sexes, aged between 18 and 65 years with ASA Grade I and II, with normal obvious anatomy scheduled to undergo elective major surgery of the upper limb below the midarm, were selected for this new lateral approach of brachial plexus block. A well-explained written consent was obtained on the hospital consent form, from all the patients.

All the patients were kept nil orally for at least 6 hours before the procedure. Infusion of Ringer lactate fluid was started with 20 G IV cannula. Premedication of inj. Glycopyrrolate 0.2 mg IV slow was given to all the patients before the start of the anaesthesia in Operation Theatre.

All blocks were performed according to a standardized procedure using a nerve stimulator (NSML-100^®^ INMED Equipments Private limited, Vadodara, India) and a 19-G × 60 mm stimulation cannula (Locoplex^®^ set, Vygon – Gurugaon (Haryana), India). The electrical current of the nerve stimulator was initially set at 2.5 mA with a stimulation frequency of 2 Hz and pulse duration of 0.1 msec.

The patient was made to lie supine with head turned to opposite side and arm pulled down gently. A small pillow or folded sheet was placed below the shoulder at interscapular area to make the field more prominent.

**Figure 1:**
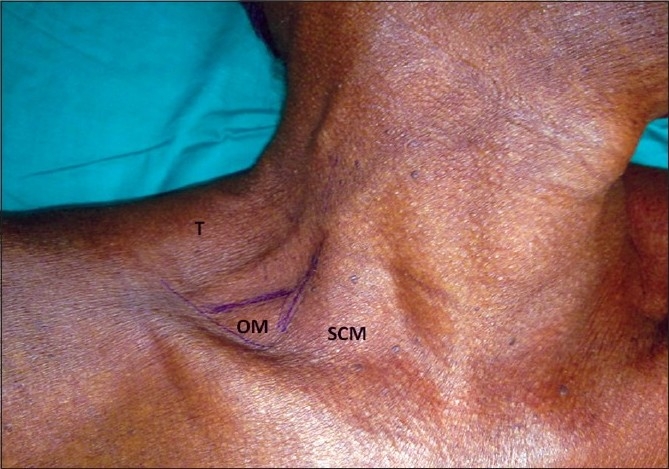
Surface landmark for lateral approach. SCM = Sternocledomastoid muscle, OM = Omohyoid muscle, T= Trapezius

**Figure 2:**
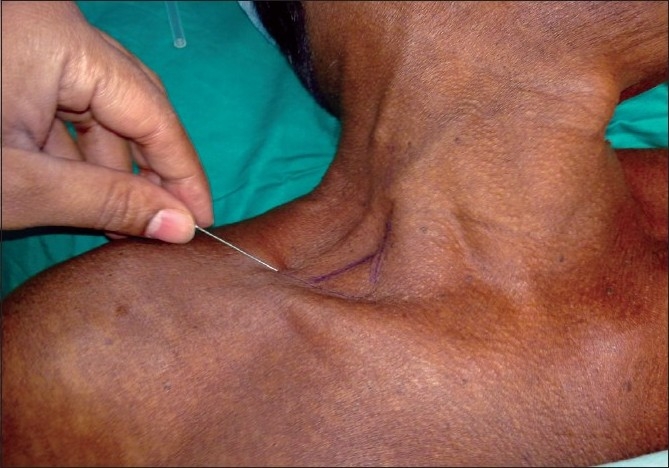
Entry point for lateral approach right side

**Figure 3:**
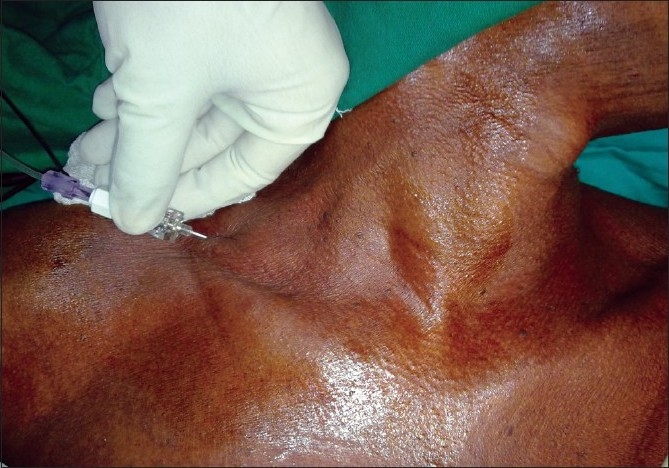
Nerve stimulation cannula in place with drug injecting

**Figure 4:**
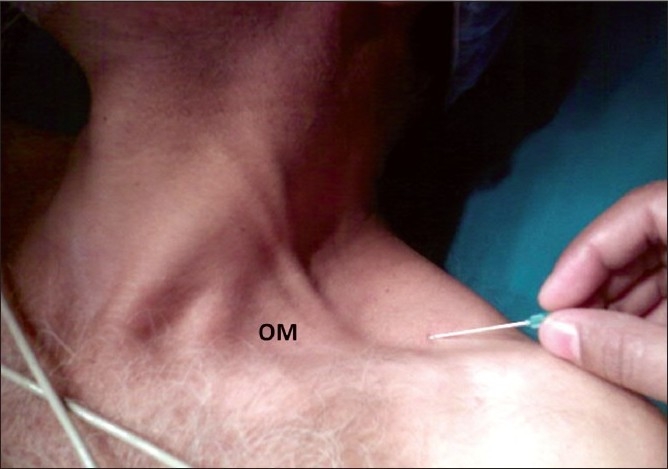
Entry point for lateral approach in the left side

**Figure 5:**
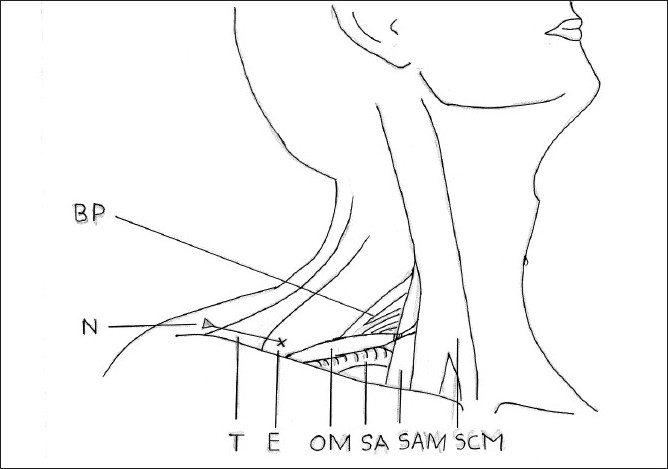
Line diagram showing lateral approch. SCM = Sternocledomastoid muscle, SAM = Sclaneus anterior muscle, SA= Subclavian artery, OM = omohyoid muscle, T= Trapezius, N= direction of needle, E= Entry point of the needle, BP= Brachial plexus

The insertion point for this lateral approach is 1 cm above the clavicle at a junction of inner two-third and outer one-third of the clavicle. The point is about 1 cm medial to border of trapazius muscle. The path is behind the omohyoid muscle and parallel to clavicle in the interscalene plane between anterior scalene and medial scalene muscle. The omohyoid muscle can be identified by rolling the index finger in the posterior triangle of the neck in normal built patients though it is not obvious in all cases. After skin disinfection and sterile covering, an intradermal wheal was raised with 1% lignocaine at the entry point. With anaesthesiologist standing at the head end, slightly toward the side, stimulation cannula was inserted through the wheal directed medially and toward the plane of the interscalene space at an angle of 20° to the skin, parallel to clavicle deep to the external jugular vein. Contraction of the forearm muscles or biceps was obtained at an electrical intensity of 0.4-0.6 mA. If stimulation does not appear and rib is contacted, the needle is walked off anterior. Once the nerve plexus is located, an assistant administered a mixture of 15 ml lignocaine 1.5% and 15 ml of bupivacaine 0.325%slowly after negative aspiration. All the patients had pressure paraesthesia during drug deposition. A gentle pressure at the area was given to make uniform spread. All the patients were given inj. Midazolam 1 mg and inj. Pentazocine 30 mg IV for sedation after successful block [Figures 1–5].

### Assessment of sensory and motor block

Immediately after the injection of drug, a successful block was defined as the absence of cold perception and response to pinch over the hand (sensory block), and paresis of the upper arm was tested as the inability to lift or abduct the forearm (motor block). Additionally in case of a fracture, the upper limb had to be pain free during passive movement for surgical preparation and positioning. Onset of sensory block and motor block was observed every 2 minutes, and time of first patient’s call for supplement analgesia and time of hand lifting against the gravity were noted at a 15-minutes interval after the surgery. If required, intermittent doses of inj. Propofol (0.5 mg/kg) IV was given to supplement the anaesthesia. When more than 50 mg of Inj. propofol was needed for continuation of surgery, then block was considered inadequate. Oxygen with ventimask was given to those who required propofol. Success and complication rate were calculated in percentage. Average onset and duration of sensory and motor block were calculated as mean + SD, and percentage with the software window office excel^®^.

## RESULTS

The age range of the 82 patients was between 18 and 65 years. Different indications for surgeries were lateral condyle humerus fixation in 22, humeral lower end surgery in 15, forearm bone fracture surgery in 20, hand surgery in 05 and A-V fistula formation with graft in 20 patients. In 75 out of 82 patients, the block resulted in successful intraoperative anaesthesia and 62 patients did not require any supplemental medications. But 15 patients required Inj. Propofol 50mg or less for skin incision only. Seven patients (8%) required more than 50 mg of inj propofol IV in addition to the block, for complete intraoperative anaesthesia.

### Sensory block

Majority of the patients had pain relief immediately after injection of drug. Average time for complete analgesia was 7.61 ± 2.82 minutes (mean ± SD). Duration of analgesia was 2-12 hours as observed by first patient’s call for supplement analgesia. Ten patients (12%) complained about tourniquet pressure pain before 120 minutes but surgery could be performed after deflation of the cuff.

### Motor loss

Average onset time for motor loss was 11.70 ± 2.50 minutes (mean ± SD); complete motor loss was present in 74 (90%) cases. Few patients moved the hand especially fingers initially but were unable to lift or abduct the arm. Average duration of motor block was 127.87 ± 14.57 minutes (mean ± SD) as observed by hand lifting against the gravity. In eight cases motor block persisted for more than 8 hours and recovered within 24 hours.

### Complications

Vessel puncture was encountered in 15% of cases in the first half and in 5% of the cases in the later half of the study and in a total of 20 (24%) cases during the procedure, but block could be performed successfully in these patients once pressure stopped the bleeding. None of the patients experienced respiratory distress or a decrease in oxygen saturat ion, pleural puncture, pneumothorax or any other cardio-respiratory side effects after the plexus block. No additional serious regional or systemic side effects or complications were observed.

## DISCUSSION

Various approaches have been described for brachial plexus block, namely, supraclavicular, interscalenous, infraclavicular, axillary and transscalene[3] routes, in search of high success rate and less complication rate. Supraclavicular technique is considered to be technically easy, associated with less serious complications but varying success rate. The divisions of the brachial plexus lie posterior, cephalic, and lateral to the subclavian artery, as they course over the first rib[4] offering a consistent and valuable anatomic relationship during placement of supraclavicular blocks.

In the present study, the block is performed where the brachial plexus is presented most compactly at the proximal division or trunk level. This compactness may explain the block’s historic reputation of providing short latency and the most complete and reliable anaesthesia for upper extremity surgery.[5] In this lateral approach, the needle passes from lateral to medial side at an angle of 20 to skin and parallel to clavicle. Once the needle meets the nerves of brachial plexus, it stimulates muscles contractions or elicits paraesthesia and then reaches to the other structures, hence chances of cervical and thoracic epidural blockade, total spinal anaesthesia, inadvertent injection into the vertebral artery, Horner syndrome and an incidence of recurrent laryngeal nerve blockade are very remote.

The incidence of vessels puncture is 15% in the first halfand 5% in the later half of the study; probably with more experience this complication may be reduced. Dr. Kothari[2] has described 8% incidence of vessels puncture. In this approach, needle is directed parallel to clavicle and not inward and downward toward inlet, and the incidence of pneumothorax is nil.[1] In our study, nonedeveloped pneumothorax. Brand and Pepper[6] injected local anaesthetic agent by Murphy’s supraclavicular route, but had 6.1% incidence of pneumothorax. Moore[7] described 1.5% incidence of pneumothorax. No patient developed Horner’s syndrome or recurrent laryngeal nerve blockade, while Pham Dang *et al*,[8] observed asymptomatic phrenic nerve paralysis (60%), Horner’s syndrome (10%) and transient recurrent nerve paralysis (1.5%). Dupre *et al*,[9] and Hempel *et al*,[1] also reported Horner’s Syndrome in 9 and 47% cases in their studies, respectively. Kumar *et al*,[10] and Ross[11] reported epidural and subdural blockade due to widespread distribution of anaesthetic agent with interscalenous route.

In lateral approach, placing needle parallel to the course of brachial plexus and near the most compact plexus of nerves of plexus, results in higher success rate (92%) in our study. Dr. Kothari[1] achieved a success rate of 98%. Moore *et al*., and Dupre *et al*., had failure rates of 8 and 11%, respectively. Brand and Papper had a success rate of 84.4%. The success rate was 85.2% with transscalene approach.[2] Pham Dang *et al*.,[7] observed satisfactory anaesthesia in 93% of the cases.

## CONCLUSION

It can be concluded that supraclavicular brachial plexus block by lateral approach is associated with minimal adverse effects in comparison to any other supraclavicular approach and is more effective with higher success rate also.
